# Central Sensitivity Symptoms and Autistic Traits in Autistic and Non‐Autistic Adults

**DOI:** 10.1002/aur.3297

**Published:** 2025-02-06

**Authors:** Sarah Grant, Sam Norton, Rosa A. Hoekstra

**Affiliations:** ^1^ Department of Psychology, Institute of Psychiatry, Psychology and Neuroscience King's College London London UK

**Keywords:** autism, central sensitisation, chronic pain, fatigue, fibromyalgia, sensory processing, sensory sensitivity

## Abstract

Central sensitivity syndromes (CSSs) are a group of health conditions thought to include an underlying sensitisation of the central nervous system. Evidence suggests autistic adults experience poorer physical health than the general population and are more likely to have a CSS. This study examined CSS diagnoses and symptoms in autistic and non‐autistic adults, to determine whether CSS symptoms were related to autistic traits, mental health, sensory sensitivity, age or gender. Participants included 534 adults with clinical diagnoses of autism, CSS, both diagnoses or neither (i.e., comparison group), who were recruited through social media, support groups and institutional affiliations. Participants completed online self‐report validated questionnaires, including the Autism Spectrum Quotient (AQ), Central Sensitization Inventory (CSI), Sensory Perception Quotient (SPQ), and the PHQ‐9 and GAD‐7. Autistic people without a diagnosed CSS reported significantly more CSS symptoms than the comparison group, with a mean score above the clinical cut‐off. Non‐autistic participants with a CSS had significantly more autistic traits than the comparison group. Autistic people with a CSS reported the most sensory sensitivity, with autism only and CSS only groups reporting similar levels of sensory sensitivity and all diagnostic groups reporting more sensory sensitivity than the comparison group. Sensory sensitivity, anxiety, autistic traits, age and gender were all significant predictors of CSS symptoms. The overlap in symptoms between autistic individuals and those with CSS suggests diagnostic overshadowing and possible under‐diagnosis or misdiagnosis. Furthermore, these symptoms may exacerbate or mask one another. Notwithstanding potential limitations of representativeness and selection bias, increased awareness of the association between autistic traits and CSS symptoms is important for clinicians to improve diagnostic accuracy and treatment.

**Abbreviations**
AQ: Autism Spectrum Quotient
CSI: Central Sensitization Inventory
CSS: Central Sensitivity Syndromes
FMS: Fibromyalgia Syndrome
GAD‐7: Generalized Anxiety Disorder Assessment
IBS: Irritable Bowel Syndrome
MCS: Multiple Chemical Sensitivity Syndrome
ME/CFS: Myalgic Encephalomyelitis/Chronic Fatigue Syndrome
PHQ‐9: Patient Health Questionnaire 9
PTSD: Post‐Traumatic Stress Disorder
RLS: Restless Legs Syndrome
SPQ: Sensory Perception Quotient
TMJ: Temporomandibular Joint Disorder

**Summary**

- Research has shown that autistic people are more vulnerable to physical ill health than non‐autistic people, including chronic health conditions known as “central sensitivity syndromes” (CSSs), including fibromyalgia, irritable bowel syndrome (IBS), migraine, and myalgic encephalomyelitis (ME/CFS). These conditions are all though to include central sensitisation as a core component, that is a state of “hyperexcitation” of the central nervous system (CNS) in which sensory input, particularly pain, is amplified. This study explored the relationship between autistic traits, CSS symptoms, sensory sensitivity and anxiety across four different groups: autistic people without a diagnosed CSS, non‐autistic people with a CSS, autistic people with a CSS, and those without a clinical diagnosis of either (comparison group). Autistic people without a CSS reported significantly more CSS symptoms, such as fatigue, pain and brain fog, than the comparison group, with many scoring in a range usually seen in diagnosed CSS patients. Non‐autistic participants with a CSS had significantly more autistic traits than the comparison group. Autistic people with a diagnosed CSS reported the most sensory sensitivity, with autism only and CSS only groups reporting similar levels of sensory difficulties and all diagnostic groups reporting more sensory sensitivity than the undiagnosed comparison group. These findings suggest commonalities between autism and CSSs and a potential for diagnostic overshadowing to occur, where one diagnosis may be missed in people who already have the other diagnosis. These findings can also inform clinical and diagnostic practice, for example by encouraging clinicians to consider screening autistic people for CSS symptoms and vice versa.

## 1 Background

Central sensitisation was originally described by Clifford Woolf (Woolf 2011) and can be described as a state of “hyperexcitation” of the central nervous system (CNS) in which sensory input is amplified, particularly in relation to pain (Meeus and Nijs 2007). This phenomenon is associated with a number of chronic health conditions, including fibromyalgia (FMS), irritable bowel syndrome (IBS), and myalgic encephalomyelitis (ME/CFS), which are often grouped together as “central sensitivity syndromes” (CSSs) (Mayer et al. 2012; Yunus 2008) or “chronic overlapping pain conditions” (COPC) (Gatchel and Neblett 2018). Whilst the focus of research into central sensitisation has been on pain, central sensitisation is associated with amplification across many different sensory domains, such that most CSS patients also report sensory hypersensitivity (Dorris et al. 2022; Suhnan, Finch, and Drummond 2017; Williams 2018).

Central sensitisation can be difficult to predict, diagnose and treat (Nijs et al. 2021; Woolf 2018) and its development is complex and multifactorial (Meeus and Nijs 2007). Research into risk factors is consequently challenging and very wide ranging, with studies showing causes and associations ranging from genetics (Castori et al. 2011; Eccles et al. 2021) to psychosocial aspects including stress (Crofford 1996, 2015; Van Houdenhove and Egle 2004) and trauma (Carmassi et al. 2017; Chandan et al. 2021; McKernan et al. 2019; Walker et al. 1997). CSSs are also related to gender, with women much more likely to experience CSS symptoms or receive a diagnosis than men (Creed 2022; Lawrence et al. 2008). Whilst some of the association with gender appears to relate to biological/hormonal differences (Lim et al. 2020; Nijs et al. 2021), stigma and diagnostic bias also appear to play a role, with clinical knowledge of a gender difference leading to dismissal of symptoms and underdiagnosis of CSS in men (Muraleetharan et al. 2018).

Some of the primary symptoms associated with CSSs are also frequently experienced by autistic and otherwise neurodivergent people; these can include sleep problems and fatigue, sensory hypersensitivity, and cognitive processing difficulties (Sala et al. 2020). Autistic people are known to be more likely to experience chronic ill health than the general population (Croen et al. 2015; Ward et al. 2023; Weir et al. 2021) and there is some evidence to suggest that chronic pain (Lipsker et al. 2018; Whitney and Shapiro 2019) and somatic symptoms (Williams 2021) might be more common in the autistic community. There is also an established link between autism and joint hypermobility, which, along with dysautonomia, frequently underlies CSS diagnoses (Csecs et al. 2022; Ryan et al. 2023). In addition, separate pain research has also indicated that sensory sensitivity could be a risk factor for developing chronic pain (Bar‐Shalita et al. 2019; Wang et al. 2020), and it has been proposed that autistic people could have a pronociceptive pain modulation profile due to an excitatory/inhibitory synaptic imbalance (Hoffman et al. 2023), or in other words, autistic people may be physiologically more sensitive to pain, and therefore more vulnerable to pain chronification. Recent reviews on autism and pain also highlight the many ways in which the autistic pain experience may differ from the general population, including pain processing, pain awareness and interoception, and social aspects of pain such as expression and communication, and suggest that all of these facets may contribute to a greater vulnerability to chronic pain including CSSs (Bogdanova et al. 2022; Moore 2015).

In line with the theory that autistic people may be more vulnerable to pain chronification (Hoffman et al. 2023), we found in a recent study that 60% of autistic adults had CSS symptoms higher than the clinical cut‐off on a commonly used measure of central sensitisation, the Central Sensitization Inventory (Grant et al. 2022; Neblett et al. 2014) whilst 21% reported a formal clinical diagnosis of a CSS. These results suggested that either CSSs are common but underdiagnosed in the autistic community, or that there is a large crossover of symptomology that has not been fully explored. Further studies have supported a link between autism and CSS symptoms (Ryan et al. 2023; Ward et al. 2023). Understanding the relationship between autism and CSS is important both for clinical practice, where it could provide new directions for diagnostic screening or treatments, and for a diverse range of research areas, including neuroscience, psychology, sociology and health.

This study aimed to extend our previous study, which focused on autistic adults only, to explore whether the same patterns and relationships could be identified in a wider group of participants including people with CSS and a comparison group. We postulated that, mirroring our previous study, higher scores on the Central Sensitization Inventory (CSI) would be associated with more autistic traits, greater sensory sensitivity and higher anxiety and depression scores, and that women would report greater sensory sensitivity and more CSS symptoms than men. We also hypothesized that autistic people with a diagnosed CSS would have greater sensory sensitivity and CSS symptoms than CSS only or autism only participants, and that all three clinical groups would report more difficulties in each area than a comparison group.

## 2 Methods

### 2.1 Participants

Participants were recruited through a variety of sources, including the King's College London internal recruitment bulletins, social media including Twitter and Facebook, support charities such as Fibromyalgia Action UK and the National Autistic Society, and the Autism Research Centre participant database at the University of Cambridge. Participants aged 18 years or over were invited to complete an online questionnaire hosted on Qualtrics and compensated for their participation through prize draw entry to win one of five £50 Amazon vouchers. Participants were also provided with sum scores and an explanation of each questionnaire at the end of the survey. All data collection was online in English and based on self‐report. All participants provided written informed consent. The study was approved by the Psychiatry, Nursing and Midwifery Research Ethics subcommittee at King's College London (HR‐18/19–8634 & RESCM‐22/23–8634).

### 2.2 Data Validation

The raw survey data was cleaned to remove participants who had completed less than 80% of the individual questionnaires within the overall survey, or who had not provided full consent. The data were also checked for duplicate entries, for uniform questionnaire answers (e.g., all “A's or all B's”) and for unrealistic completion times that indicated participants could not have read or understood the questions properly. Most participants also provided contact emails that could be checked through to minimize duplicates and ensure validity. The calculation of sum scores during the questionnaire process also allowed the researchers to double check the entries for any participants with very high or very low scores, to minimize the possibility of “fake” or “professional” participants (Godinho, Schell, and Cunningham 2020). This process reduced the data from 668 participants to 534.

### 2.3 Measures

#### 2.3.1 Central Sensitisation

The Central Sensitization Inventory (Mayer et al. 2012) was utilized in this study due to its validity and reliability as a self‐report measuring symptoms of central sensitisation (Cuesta‐Vargas et al. 2018; Neblett et al. 2014). Each item in Part A is measured using 25 items on a five‐point Likert scale ranging from 0 “never” to 4 “always.” A cut‐off score of 40 on Part A was reported to best distinguish between CSS and non‐CSS patients (Neblett et al. 2014). The Dutch version of the CSI (Kregel et al. 2016) was demonstrated in our 2022 study to be valid in an autistic population (Grant et al. 2022). In the current sample the Cronbach's *α* = of the scale is 0.95 indicating excellent internal consistency. Part B contains a list of CSS diagnoses and related disorders. All health conditions listed in Part B of the CSI—restless legs syndrome (RLS), fibromyalgia syndrome (FMS), myalgic encephalomyelitis/chronic fatigue syndrome (ME/CFS), irritable bowel syndrome (IBS), migraine/tension headaches, multiple chemical sensitivity (MCS) and temporomandibular joint disorder (TMJD)—were included in the online survey, with participants able to select them as either a formal clinical diagnosis or as a suspected/self‐diagnosis. Interstitial cystitis (IC) was also included, as this condition is thought to come under the CSS umbrella and was included in an assessment of the reliability and clinical significance of the CSI (Neblett et al. 2014). Participants with one or more formal CSS diagnoses were flagged as “diagnosed CSS” with all others flagged as not diagnosed.

#### 2.3.2 Sensory Sensitivity

The Sensory Perception Quotient (SPQ) is a 35‐item measure that was developed to assess sensory sensitivity in adults with and without autism. This measure shows good internal consistency and validity (Tavassoli, Hoekstra, and Baron‐Cohen 2014; Taylor et al. 2020; Weiland et al. 2020) with a Cronbach's *α* = 0.94 in this sample. The SPQ is assessed across five sensory modalities, on a Likert scale ranging from 0 “strongly agree” to 3 “strongly disagree.” A low SPQ score would therefore indicate higher sensory sensitivity than a high score.

#### 2.3.3 Autistic Traits

Autistic traits were measured using the 50 item Autism Spectrum Quotient or AQ (Baron‐Cohen et al. 2001). Items are scored on a four‐point Likert scale ranging from 1 “definitely agree” to 4 ‘definitely disagree’. 24 of the 50 items are reverse scored where “agree” responses are characteristic for autism. In order to capture the full range of autism spectrum traits, the AQ scores for this study were calculated using the full Likert values, producing a minimum AQ score of 50 and a maximum of 200. A score greater than 145 is considered to be specific for autistic people (Hoekstra et al. 2008), and we have therefore used this score as a cut‐off in the analyses in the current paper. It should be noted that, whilst a cut‐off score is necessary for analytic purposes, the AQ is a trait measure, and should not be considered diagnostically sensitive. The Autism Spectrum Quotient has been extensively evaluated and is widely used, however there is variation in the research support for its reliability, sensitivity and specificity (Ashwood et al. 2016; Conner, Cramer, and McGonigle 2019; Hoekstra et al. 2011; Jia, Steelman, and Jia 2019; Murray et al. 2017). In this sample, a Cronbach's α of 0.96 indicates excellent internal consistency.

#### 2.3.4 Depression

The PHQ‐9 is a widely used brief measure designed to capture depression severity in the general population (Kroenke, Spitzer, and Williams 2001). Each of the nine Likert scale items ranges from 0 “Not at all” to 3 “Nearly every day,” with a separate question also asking how much these problems affect daily life. A higher score on the PHQ‐9 indicates greater symptom severity. The PHQ‐9 has been utilized and tested in many different cultures and patient populations, and has been demonstrated to be a reliable diagnostic tool (Costantini et al. 2021; Kroenke et al. 2010; Wittkampf et al. 2007). Anxiety.

The GAD‐7 is a brief measure designed in the same format as the PHQ‐9 to assess generalized anxiety symptoms (Spitzer et al. 2006). As for the PHQ, each of the seven Likert scale items range from 0 “Not at all” to 3 “Nearly every day.” A separate question scored from 0 “Not difficult at all” to 3 “Extremely difficult” is designed to assess how much anxiety symptoms are affecting daily life. A higher score on the GAD‐7 indicates greater symptom severity. As per the PHQ‐9, the GAD‐7 has been globally utilized and tested, and has been found to have good validity and reliability (Kroenke et al. 2010; Löwe et al. 2008).

### 2.4 Statistical Analyses

To investigate group differences, the cleaned and validated data were divided into four participant groups – “autism only” for those participants with a formal clinical diagnosis of autism and no CSS diagnosis, “CSS only” for those participants with a formal CSS diagnosis only, “autism and CSS” for participants with a formal clinical diagnosis of both autism and one or more CSS, and “comparison,” who had neither an autism nor a CSS diagnosis. The total sample size at this stage was *n* = 473. Gender differences were first evaluated using ANOVA. Group differences were then evaluated using ANCOVA, including gender and age as covariates. To ensure meaningful group comparisons, any participants that suspected they might be autistic and/or have a CSS but did not have a formal diagnosis of either were excluded from the comparison group. Participants with a formal autism diagnosis that indicated they suspected they had a CSS but did not have a formal diagnosis, were placed in the autism only group. Likewise, those with a formal CSS diagnosis and suspected autism were placed in the CSS only group. The dual diagnosis group comprised participants with formal clinical diagnoses for both autism and one or more CSS. Participants who indicated a gender other than male or female were excluded due to insufficient power, but there were no further exclusions after this. Where relevant, analyses were corrected for multiple testing using using Dunn‐Šidák correction to minimize the chance of type I errors in this exploratory research.

A four‐stage hierarchical regression analysis was then used with the full sample, minus those that had indicated “other” gender (*n* = 519), to explore whether autistic traits, sensory sensitivity and anxiety might significantly predict CSS symptoms. Age and gender were included in stage one as controls, since these variables had been identified to be significant predictors of CSS symptoms (Grant et al. 2022; Mayer et al. 2012), and then each construct was added in a separate stage to explore their effect on the variance in CSI scores.

A path analysis was conducted using the model developed in our earlier study that included autistic adults only (Grant et al. 2022), to establish whether sensory sensitivity or anxiety might mediate the relationship between autistic traits and CSS symptoms. This model was also applied to the larger sample available, *n* = 519. As this model incorporates measures that have all been validated in the general population, it was felt that using this model with a more diverse cohort might provide important insights into how the relationships between the measures might work, and whether the pattern identified in autistic adults still applied. The previous path model utilized the HADS‐A measure for anxiety (Zigmond and Snaith 1983) and, as anxiety and depression were highly correlated, anxiety alone was used as this had a stronger correlation with CSS symptoms. Therefore, in this version of the path analysis, the GAD‐7 is used as the anxiety measure. The path analysis was conducted using MPlus version 8.1 (Muthén and Muthén 2017) with all other analyses conducted using SPSS 29.0 (IBM 2023).

## 3 Results

### 3.1 Descriptive Statistics

The initial sample comprised 534 adults (91 men, 428 women, 15 other), and the demographics are laid out in Table 1. In this sample, men (Mean = 45.2 SD = 16.53) were significantly older than women (Mean = 38.0 SD = 12.55) and individuals with other gender expressions (Mean = 35.0 SD = 10.74). The majority of participants were well educated, with 68.2% sample holding at least an undergraduate degree, and they were also predominantly from Western countries (see Table 1 for breakdown).

**TABLE 1:** Sample demographics.

		Men	Women	Other (excluded)	Total
Number of participants		91	428	15	534
Age	Mean (SD)	45.2 (16.53)	38.0 (12.55)	35.0 (10.74)	39.2 (13.53)
	Minimum age	18	18	21	18
	Maximum age	73	90	54	90
Education	No education or primary school only	0	6	0	1.12%
	Education up to age 16	10	28	0	7.12%
	Education up to age 18	25	98	3	23.60%
	Undergraduate degree	31	166	7	38.20%
	Postgraduate degree	25	130	5	29.96%
Location	United Kingdom	76	359	10	83.33%
	USA	7	22	3	5.99%
	Australia	2	16	0	3.37%
	Europe	4	23	1	5.24%
	Other	2	8	1	2.06%
CSS Diagnosis	No clinical diagnosis	53	122	3	33.33%
	One clinical CSS diagnosis	24	88	3	21.54%
	Multiple clinical CSS diagnoses	14	218	9	45.13%
Specific CSS Diagnoses^a^	ME/chronic fatigue syndrome (ME/CFS)	8	98	4	20.60%
	Fibromyalgia (FMS)	14	214	6	43.82%
	Migraine	14	144	8	31.09%
	Irritable bowel syndrome (IBS)	16	161	7	34.46%
	Multiple chemical sensitivity (MCS)	1	24	0	4.68%
	Restless legs syndrome (RLS)	4	63	0	12.55%
	Interstitial cystitis (IC)	0	12	1	2.43%
	Temporomandibular Joint disorder (TMJD)	2	74	4	14.98%

^a^ Many participants had more than one CSS diagnosis, and therefore the totals represented here will add up to more than the number of participants in the study.

Out of 534 participants, 356 participants (67% of the sample) had at least one formally diagnosed CSS. 115 (32%) of the 356 participants had only one clinical CSS diagnosis and the remaining 241 had more than one. 93% of participants with a diagnosed CSS scored at or above the clinical cut‐off of 40 on the CSI. 130 participants (24%) had a formal diagnosis of autism. 86% of participants with an autism diagnosis scored at or above the suggested cut‐off score of 145 (Hoekstra et al. 2008) on the AQ.

All subsequent analyses focussed on men and women participants only as there were too few participants in the “other” gender group (*N* = 15).

### 3.2 Group Differences

Group differences were explored by restricting the data to four clearly delineated groups autism only (*n* = 31), CSS only (*n* = 257), both conditions (*n* = 87), and a comparison group who have neither condition nor a suspicion they may be autistic or have CSS, (*n* = 98), leaving a total *n* = 473.

The mean scores for each measure were analyzed using one way ANOVA to determine whether there were any group differences in gender. Women scored significantly lower on the AQ than men (*f* (1,471) = 7.65 *p* = 0.006), significantly higher on the CSI (*f* (1,471) = 26.44 *p* < 0.001) and significantly higher on the PHQ (*f* (1,471) = 6.26 *p* = 0.013).

Table 2 presents the group differences on the main measures of interest, controlling for age and gender. There were significant differences on every measure between the four participant groups. Pairwise comparisons to the comparison group have also been included in the table.

**TABLE 2:** Comparisons between different clinical groups with gender and age as covariates.

		Participant group			
	Measure	Comparison	CSS only	Autism only	CSS and autism
	*N*	98	257	31	87
CSI	Estimated marginal mean (SE)	32.56 (1.58)	66.81 (0.96)	52.00 (2.75)	66.74 (1.61)
	Mean dif. (SMD) versus comparison	—	−34.24*	−19.44*	−34.18*
	F Test	*f* (3,467) = 119.76 *p* < 0.001***			
	Covariates [gender, age]	[*f* (1,467) = 8.91 *p* = 0.003**, *f* (1,467) = 0.03 *p* = 0.87]			
AQ	Estimated marginal mean (SE)	107.48 (2.402)	126.31 (1.46)	154.70 (4.18)	161.92 (2.44)
	Mean dif. (SMD) versus comparison	—	−18.83*	−47.22*	−54.44*
	F test	*f* (3,467) = 99.34 *p* < 0.001***			
	Covariates [gender, age]	[*f* (1,467) = 4.53 *p* = 0.03, *f* (1,467) = 0.55 *p* = 0.46]			
SPQ	Estimated marginal mean (SE)	52.15 (1.661)	38.61 (1.01)	38.53 (2.89)	31.78 (1.69)
	Mean dif. (SMD) versus comparison	—	13.55*	13.63*	20.38*
	F test	*f* (3,467) = 26.04 *p* < 0.001***			
	Covariates [gender, age]	[*f* (1,467) = 2.31 *p* = 0.13, *f* (1,467) = 1.52 *p* = 0.22]			
PHQ	Estimated marginal mean (SE)	6.29 (0.676)	13.76 (0.41)	10.34 (1.18)	14.95 (0.69)
	Mean Dif. (SMD) versus comparison	—	−7.48*	−4.05*	−8.67*
	F test	*f* (3,467) = 35.20 *p* < 0.001***			
	Covariates [gender, age]	[*f* (1,467) = 0.38 *p* = 0.54, *f* (1,467) = 2.04 *p* = 0.15]			
GAD	Estimated marginal mean (SE)	4.05 (0.605)	9.85 (0.366)	8.87 (1.052)	11.59 (0.616)
	Mean dif. (SMD) versus comparison	—	−5.796*	−4.817*	−7.536*
	F test	*f* (3,467) = 29.27 *p < 0*.001***			
	Covariates [gender, age]	[*f* (1,467) = 0.008 *p* = 0.93, *f* (1,467) = 10.29 *p* = 0.001**]			

*Note*: Group differences significant at 0.05 level with Bonferroni adjustment for multiple comparisons.

Abbreviations: CSI = central sensitization inventory; SPQ = sensory perception quotient; AQ = autism spectrum quotient—short; PHQ = patient heath questionnaire 9—depression; GAD—generalized anxiety Disorder assessment 7; SMD = standardized mean difference.

* *p* < 0.05.

** *p* < 0.01.

*** *p* < 0.001.

**FIGURE 1:**
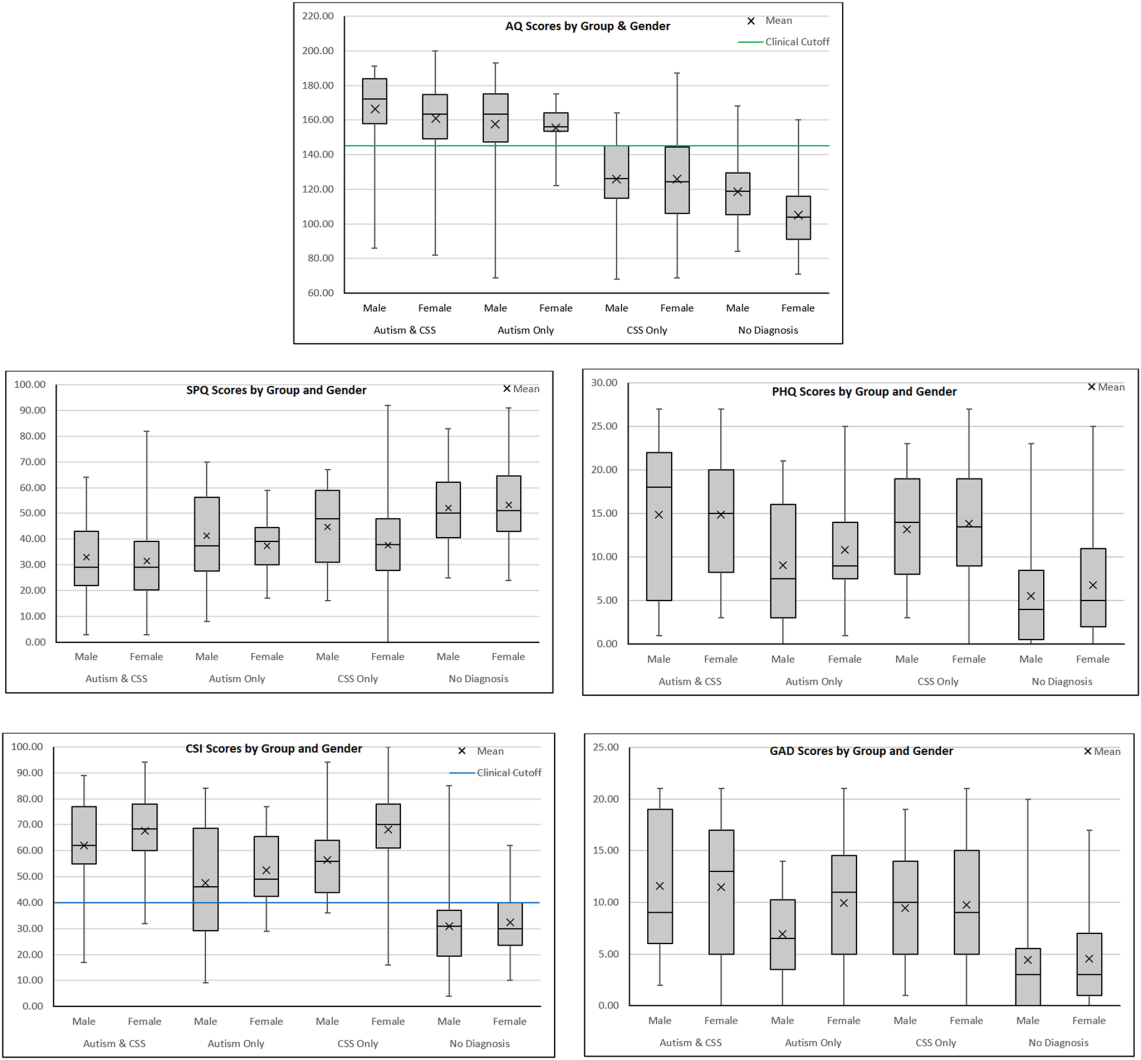
Group differences for main measures—box and whisker plots.

Pairwise comparisons using the Dunn‐Šidák correction demonstrated several notable group differences, which are visualized in Figure 1. Firstly, autistic people without a CSS diagnosis scored significantly higher on the CSI than the comparison group, with an estimated marginal mean score in the autistic sample (52.00) well above the clinical cut‐off of 40. The CSI scores between autistic and non‐autistic people with a CSS diagnosis were not significantly different, however (66.81 vs. 66.74; *p* = 1.00). On the AQ, as expected diagnosed autistic groups scored significantly higher than non‐autistic groups (see Table 1) and there was no significant difference in AQ scores between autistic people with and without a CSS (161.92 vs. 154.70; *p* = 0.578). However, non‐autistic participants with a CSS scored significantly higher on the AQ than the comparison group (126.31 vs. 107.48; *p* < 0.001). When analyzing mean differences for sensory sensitivity, all three diagnostic groups scored significantly lower than the comparison group (see Table 1). Autistic people with a CSS diagnosis also reported greater sensory sensitivity than people with just a CSS diagnosis (31.78 vs. 38.61 *p* = 0.004) but the small mean difference between this group and those with just an autism diagnosis (38.61 vs. 38.53) was not significant (*p* = 0.232).

Lastly, there were group differences in mental health symptoms. All three clinical groups had higher depression scores than the comparison group (Table 1), but people with a CSS only diagnosis had significantly higher depression scores than those with an autism only diagnosis (13.76 vs. 10.34, *p* = 0.040). People with both diagnoses had comparable depression scores with CSS only participants (14.95 vs. 13.76, *p* = 0.596). For anxiety, all diagnostic groups were significantly more anxious than the comparison group. Autistic people were significantly more anxious than the comparison group (*p* < 0.001), but the differences between autistic only and CSS only (8.87 vs. 9.85, *p* = 0.946), autism and CSS versus CSS only (11.59 vs. 9.85, *p* = 0.092) and autism and CSS versus autistic only (11.59 vs. 8.87, *p* = 0.142) were not significant.

Group Differences for AQ Subdomains As one of the findings was that the CSS only group scored higher on the AQ than the comparison group (but with a mean below the proposed cut‐off score for autism), a post hoc analysis of subdomains of the AQ was completed, to explore whether there were different patterns of answers for different groups. The subdomains were identified from the same paper that used the enhanced scoring method utilized in this paper (Hoekstra et al. 2008). For the Social Skill subdomain, mean scores followed the same pattern as for the full AQ scores, with the autism & CSS group having the highest score (33.13), then autism only (30.58), then CSS only (26.27), and then the comparison group (20.96) where there were significant differences between all scores and the comparison group (*p* < 0.001) but not between the autism & CSS group and autism only group (*p* = 0.259). The Communication, Attention to Detail and Attention Switching subdomains also followed this same pattern, with significant differences between all groups except between the autism & CSS and autism only groups (Communication *p* = 0.705, Attention to Detail *p* = 0.877, Attention Switching *p* = 0.872). The Imagination subdomain showed a slightly different pattern, with no significant difference between the estimated marginal means for the CSS only group (21.078) and the comparison group (19.693, *p* = 0.279). Both autism groups scored significantly higher on this subdomain than the comparison group (*p* < 0.001) and, as for the full AQ, there was no significant difference between autism & CSS and autism only participants (*p* = 1.00).

### 3.3 Hierarchical Regression and Path Analysis

The relationships between the main variables were analyzed using regression and path analysis. All men and women regardless of diagnoses were included in this analysis (*n* = 519). A four‐stage hierarchical multiple regression with CSI score as the dependent variable was utilized. Stage one included age and gender, stage two included autistic traits (AQ), stage three added sensory sensitivity (SPQ) and stage four anxiety (GAD). The results are shown in Table 3.

**TABLE 3:** Hierarchical regression analysis—predictors of CSS symptoms.

	Model one			Model two			Model three		
	Beta	*t*	*p*	Beta	*t*	*p*	Beta	*t*	*p*
(Constant)		9.23	< 0.001***		−1.63	0.10		5.38	< 0.001***
Gender	0.28	6.45	< 0.001***	0.32	8.43	< 0.001***	0.22	6.99	< 0.001***
Age	0.20	4.66	< 0.001***	0.16	4.06	< 0.001***	0.15	4.97	< 0.001***
AQ score				0.45	11.85	< 0.001***	0.12	3.29	0.001**
SPQ score							−0.29	−7.99	< 0.001***
Anxiety							0.44	13.00	< 0.001***

*Note: R* ^2^ = 0.094 for Model 1: ΔR^2^ = 0.194 for Model 2 (*p* < 0.001): ΔR^2^ = 0.265 for Model 3 (*p* < 0.001).

** *p* < 0.01.

*** *p* < 0.001.

**FIGURE 2:**
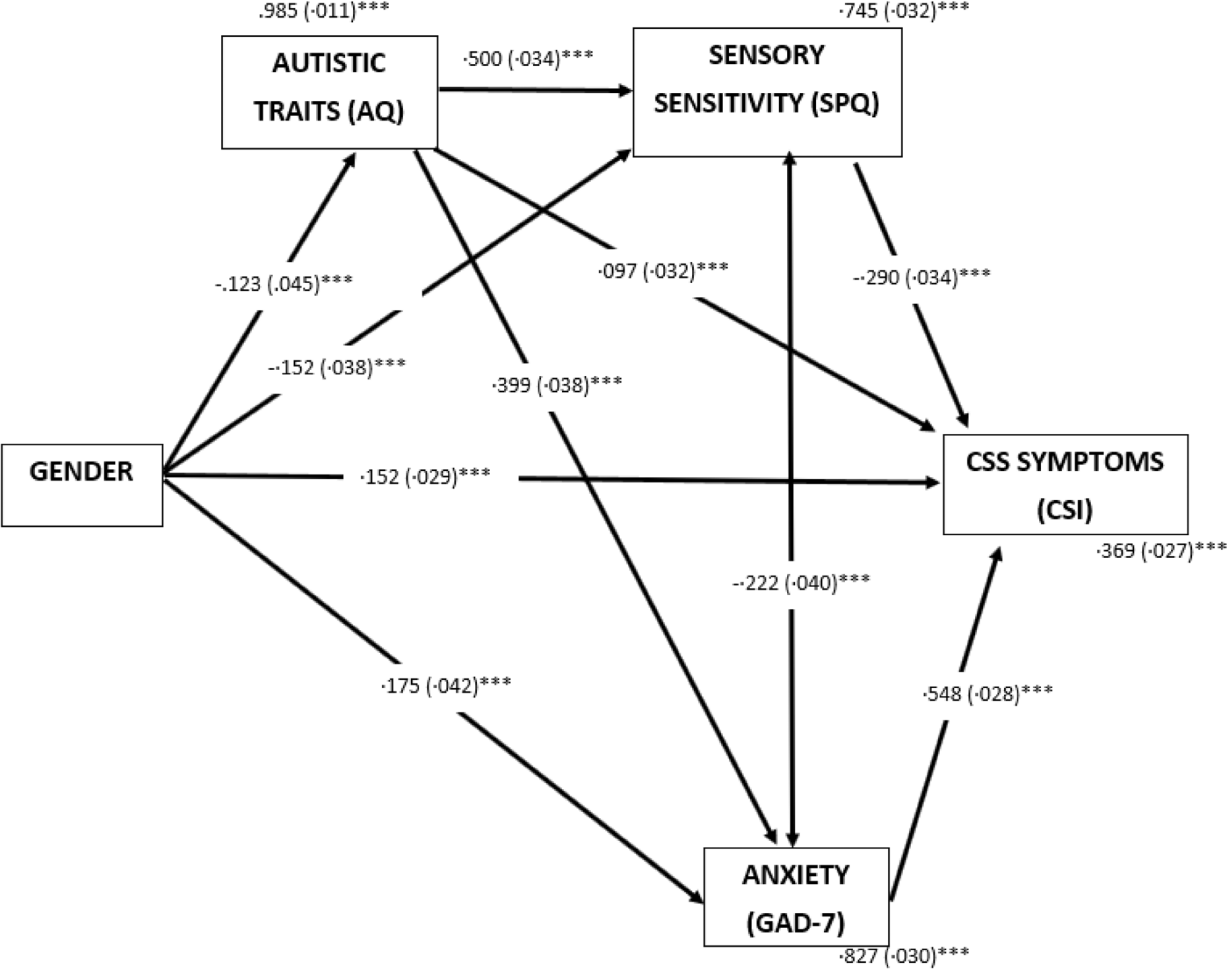
Path model exploring the relationship between main variables and CSS symptoms. Standardized path coefficients (S.E), covariates and residual variance shown. *p < 0.05; **p < 0.01; ***p < 0.001.

Stage one of the regression analysis demonstrated that gender and age were both significantly associated with CSS symptoms but only accounted for 9.4% of the variance in CSI scores. Stage two contributed significantly to the regression model, *F* (3,515) = 69.37, *p* < 0.001, with AQ scores explaining 19.4% additional variance in CSI scores. Sensory sensitivity contributed to a further 11.8% of the variance (*F* (4,514) = 87.69, *p* < 0.001) and anxiety to 14.7% of the variance (*F* (5,513) = 126.92, *p* < 0.001). In total the model accounted for 54.9% of the variance in CSI scores. Overall, the regression analysis indicated that age, gender, autistic traits, sensory sensitivity and anxiety are all significant predictors of CSS symptoms. The path model from our previous autistic adults‐only study (Figure 2) was applied to the data to explore relationships in a broader participant constituency. This model includes gender as a predictor variable, CSS symptoms as an outcome, and autistic traits, anxiety and sensory sensitivity as mediator variables, with the rationale being that gender is established early in life, with autistic traits and sensory sensitivity also present from a young age, whereas CSS symptoms are known to manifest later on. As previously, gender significantly predicted sensory sensitivity (*p* < 0.001), anxiety (*p* < 0.001), and CSS symptoms (*p* < 0.001). Autistic traits significantly predicted both sensory sensitivity (*p* < 0.001) and anxiety (*p* < 0.001). Sensory sensitivity, anxiety and autistic traits all significantly predicted CSI scores (*p* < 0.001). This differed from our previous study, where sensory sensitivity and anxiety mediated a relationship between autistic traits and CSS symptoms.

## 4 Discussion

This study built on previous research demonstrating an association between autism and CSS by comparing central sensitisation symptoms, autistic traits, sensory sensitivity, and mental health symptoms in autistic and non‐autistic adults with and without a diagnosis of a central sensitivity syndrome. In this sample, we compared means on a range of self‐reported measures across four groups; adults with CSS clinical diagnosis only, adults with a formal autism diagnosis only, adults with both an autism and CSS diagnosis, and a comparison group of adults who do not have either diagnosis.

The findings of this study showed that (a) the mean CSI score for autistic people without a diagnosed CSS was still well above the clinical cut‐off (Neblett et al. 2014), suggesting that symptoms of central sensitisation are common in autism, (b) non‐autistic people with a diagnosed CSS had more autistic traits than the comparison group, (c) autistic people without a CSS, and non‐autistic people with a CSS, showed comparable levels of sensory sensitivity and (d) depression was strongly associated to CSS symptoms, and highest in autistic people with a CSS.

The finding that autistic people without a CSS experience a lot of CSS symptoms is in line with our previous study (Grant et al. 2022), which found that symptoms such as pain and fatigue are common in autistic individuals. It is also notable that the mean CSI scores for CSS only participants and participants with dual autism & CSS diagnoses were not significantly different. One of the questions around an association between autism and CSS is whether the CSI is measuring CSS symptoms in autistic people or whether the nature of the questions mean that it is capturing some other elements of autism. If this were the case, then in this study we would expect to see higher CSI scores for people with autism and a CSS diagnosis than for CSS only participants. Our observation of similar scores in the CSS only group and the dual autism and CSS group could suggest that the CSI assesses specifically CSS symptoms rather than a broader set of characteristics linked to autism. This is supported by the factor analysis of the CSI in autistic adults conducted in our previous study (Grant et al. 2022) which followed the same factor structure as that found in a pooled multi‐country sample (Cuesta‐Vargas et al. 2018). However, the strong relationship between autistic traits and CSS symptoms demonstrated in the regression analysis also suggest that there could be an alternative explanation. The non‐significant difference between these scores could be an artifact of the way the participant groups were divided strictly according to clinical diagnosis, whereby there would likely have been some participants in the CSS only group that suspected autism and/or that had higher autistic traits, and potentially then higher CSI scores as well.

Following on from this, we found that there was no significant difference in autistic traits between autistic people with and without a CSS, despite a strong relationship between autistic traits and CSS symptoms overall, as demonstrated in the regression and path analyses. As for the CSI scores, this could also be because of the way the participant groups were divided, with participants in the autism only group that suspected they might have a CSS but did not have a formal clinical diagnosis, and it may have been the case that a clearer AQ difference would have been seen if these participants had been excluded. The participant groupings are discussed further in the Limitations section. We also found that non‐autistic people with a diagnosed CSS had higher autistic traits than the comparison group, though the mean score was lower than the proposed cut‐off score for autism on this scoring version of the measure (Hoekstra et al. 2008). This finding could suggest that autism is being missed in some people with CSS. An exploration of the subdomains of the AQ showed no significant differences between groups, except for the Imagination subdomain, where autistic individuals scored significantly lower than non‐autistic participants. The analysis suggests that the pattern of answers for CSS only participants is broadly similar to the autistic participants, and the increased AQ scores in this group are likely to be related to increased autistic traits, rather than the AQ capturing elements of the CSS experience.

Significant differences in SPQ scores were also found between all three diagnostic groups compared to the comparison group, who reported higher SPQ scores and therefore much lower sensory sensitivity overall (see Table 1). Participants with both a CSS and an autism diagnosis were the most sensory sensitive, however SPQ scores between autistic participants without a CSS, and non‐autistic participants with a CSS, were comparable (38.5 vs. 38.6). This is in line with a study by Dorris et al. (2022) in which the average fibromyalgia SPQ score was similar to SPQ scores for autistic adults. This finding has important implications for CSS patients, for whom the impact of sensory differences has historically not been fully acknowledged or explored (Harriott and Schwedt 2014; Schrepf et al. 2023; Staud, Godfrey, and Robinson 2021). In addition to group differences, sensory sensitivity was indicated as a predictor of CSS symptoms. As previously hypothesized, it could be the case that the multisensory integration mechanisms responsible for the development of sensory sensitivity in chronic pain patients could also explain a relationship in the opposite direction that is, chronic pain in autistic people with sensory processing differences. Very recent research on the relationship between generalized sensory sensitivity and chronic pain supports this theory (Bar‐Shalita et al. 2019; Dixon et al. 2016; Schrepf et al. 2023; Wang et al. 2021).

As predicted, significant differences between all clinical groups and the comparison group were also found for both depression and anxiety symptoms. Symptoms of depression were also higher for CSS patients, regardless of an autism diagnosis, whilst autistic people without a CSS still reported more depression than the comparison group. This is in line with previous research showing that chronic pain (Adams and Turk 2015; Katon 2011) and autism (Croen et al. 2015; Lai, Lombardo, and Baron‐Cohen 2013) are both strongly associated with depression. Depression is also the most common mental health condition to co‐occur with chronic pain and is thought to be both an outcome and an exacerbator of pain symptoms (Adams and Turk 2015) as well as being associated with chronic illness more broadly (Clarke and Currie 2009; Gold et al. 2020). In addition, the PHQ‐9 measure used in this study includes two questions that relate to fatigue (Kroenke et al. 2010). Anxiety symptoms were significantly higher in all clinical groups compared to the comparison group, but with no significant differences between the clinical groups. Anxiety is associated with both autism and CSS (Adams and Turk 2015; Lever and Geurts 2016), so these results are not unexpected.

Whilst the analyses in this article were more focussed on clinical group differences, the gender differences demonstrated in our previous article were replicated in this sample, with women reporting more CSS and depression symptoms than men and reporting lower autistic traits overall. Lastly, as predicted, hierarchical regression analysis indicated that higher scores on the CSI were associated with greater sensory sensitivity and greater anxiety. In contrast to our previous study in an autistic only sample, there was also a clear association between autistic traits and CSS symptoms. This may be because the current sample included non‐autistic adults and therefore allowed for a greater range of AQ scores.

These findings build on our current understanding of the association between autism and central sensitisation, and the inclusion of a comparison group and CSS only participants allow for a more detailed exploration of similarities and differences between clinical groups. The results of this new paper suggest that diagnostic overshadowing might be an issue for both CSS and autism, with many autistic people displaying CSS symptoms without a CSS diagnosis, and people with CSS displaying increased autistic traits compared to a comparison group. It is possible that these issues are also gender related, since autistic women tend to be underdiagnosed (Begeer et al. 2013; Loomes, Hull, and Mandy 2017) whereas CSSs are more commonly recognized in women compared to men (Lawrence et al. 2008). Whilst there is a demonstrable overlap in physical symptoms between autism and CSSs, the results of this study suggest that the CSI and AQ are both working reliably and consistently to identify symptoms of central sensitisation and autistic traits, and that there are increased rates of central sensitivity symptoms and diagnoses in autistic adults (Ward et al. 2023). To further understand the development and diagnosis of CSSs in autistic people, longitudinal follow‐up studies that explore sensory sensitivities, CSS symptoms and mental health symptoms over time would be beneficial. In addition, qualitative studies that identify whether CSS symptoms and autism traits could exacerbate each other, or mask each other, would provide further clarity on the degree to which diagnostic overshadowing could occur.

Diagnostic overshadowing remains relevant when considering that research on the established link between autism and joint hypermobility could also explain the association between autism and CSSs. Joint hypermobility disorders and the Ehlers‐Danlos syndromes (EDS) are often found to underly CSS diagnoses (Csecs et al. 2022; Eccles et al. 2021; Sharp, Critchley, and Eccles 2021), and autistic women in particular are more likely to be diagnosed with EDS than the general population (Casanova et al. 2020; Ward et al. 2023). There is also some research suggesting that sensory sensitivity is directly related to connective tissue differences (Casanova et al. 2020; Colman et al. 2023; Csecs et al. 2022; Savage et al. 2021). More research is therefore needed to understand whether sensory sensitivity is an independent risk factor for developing a CSS (Bar‐Shalita et al. 2019), or an artifact of an underlying genetic connective tissue difference.

The relationships between autistic traits, sensory sensitivity and mental health highlighted in this and our previous study raise questions about the underlying mechanisms of a relationship between autism, central sensitisation and CSSs. Although studies of neurobiological mechanisms that may be relevant to pain in autism are limited, some research has explored pain mechanisms in autism through quantitative sensory testing (QST). A recent systematic review of some of these studies found evidence of autistic hypersensitivity to both heat and cold pain thresholds, and lower pressure pain thresholds (Vaughan et al. 2019). Lower pain pressure thresholds are also often found in central sensitisation and have in fact been suggested as a possible way of testing for the phenomenon (den Boer et al. 2021). A later QST study found that some autistic participants experienced paradoxical heat sensations and dynamic mechanical allodynia, phenomena that are not typically found in neurotypical individuals, but are associated with central sensitisation (Baron, Hans, and Dickenson 2013; Lolignier, Eijkelkamp, and Wood 2015). Related research has also found altered neural responses to sustained pain in autistic people (Failla et al. 2018) and higher pain ratings, pain anxiety and pain‐related fear, suggesting a reciprocal interaction between sensory and cognitive autistic pain experiences (Failla et al. 2020). These studies, as suggested in a recent review by (Hoffman et al. (2023)), indicate that autistic individuals could be physiologically more sensitive to pain, and more vulnerable to pain chronification.

This study also demonstrates a need for a more detailed examination of the psychosocial aspects of chronic illness, and how these may differ in autistic people. Our results show that autistic people with a CSS are more prone to depression than autistic adults without a CSS, which is problematic for a community that already experiences increased rates of mental health difficulties and suicide (Cassidy and Rodgers 2017; Hirvikoski et al. 2016; Lai, Lombardo, and Baron‐Cohen 2013). An increasing body of research has demonstrated that autistic people are more vulnerable to a broad range of chronic illness and disease (Croen et al. 2015; Rydzewska et al. 2019; Ward et al. 2023; Weir et al. 2021; Williams and Gotham 2022), and that there are also significant barriers to healthcare for autistic people (Doherty et al. 2022; Nicolaidis et al. 2015; Shaw et al. 2024), but there is very little research that examines the lived experience of autistic people with co‐occurring medical conditions, or considers psychosocial factors in the context of physical ill health, such as camouflaging (Pearson and Rose 2021), illness beliefs (de Heer, Vriezekolk, and van der Feltz‐Cornelis 2017; Moss‐Morris et al. 2002) and epistemic injustice (Blease, Carel, and Geraghty 2017; Chapman and Carel 2020). In addition, as previously discussed, there may be neurobiological factors at play that influence the association between CSSs, autism, sensory sensitivity and depressive symptoms and further research is warranted that explores associations at the neurobiological, psychological and social levels, including an exploration of CSS symptoms and diagnoses in transgender and non‐binary adults.

Lastly, there are clinical implications to this work that should be considered going forward. The finding that CSS symptoms are more common in autistic people should guide clinicians in considering autism screening for CSS patients, and CSS screening for autistic people presenting with pain or fatigue, to minimize the chances of diagnostic overshadowing. This consideration should be even more pertinent for women, in whom autism is under‐recognized (Harper et al. 2019; Tsirgiotis, Young, and Weber 2022) and CSSs more prevalent (Lawrence et al. 2008; Wolfe et al. 2018). Our results could also suggest that environmental adjustments and therapies traditionally offered to autistic people for sensory difficulties, such as occupational therapy (Pfeiffer and Kinnealey 2003; Schaaf et al. 2018), might benefit CSS patients. Future research could include the development of integrated clinical treatment plans that incorporate therapies that address sensory sensitivity and mental health difficulties, as well as pain and fatigue.

## 5 Limitations

Whilst we were able to include a large and diverse sample of participants in this study, it must be noted that recruitment for the study was attained through publication in various patient groups, including fibromyalgia support groups, the Autism Research Centre in Cambridge, and through social media. This is reflected in the participant demographics, where 67% had a diagnosed CSS, and the majority of the participants were also women, in line with previous studies showing that women are more likely to participate in online surveys (Aerny‐Perreten et al. 2015; Rubenstein and Furnier 2021). We can therefore be confident that there was some selection bias. However, the similarity of the findings of the current study with the findings reported previously in our autism‐only sample, suggest selection bias had a limited effect. The previous study used an existing volunteer register of autistic people, who completed the surveys as part of a yearly data collection wave rather than a specifically advertised study and was therefore unlikely affected by selection bias favoring participation of people with high CSS symptoms. In addition to selection bias, it is also important to acknowledge that participation in this study was limited to people that were able to self‐report in an online survey, and therefore the results may not generalize to every autistic person or CSS patient. Future studies could be conducted in participants who are already members of existing autism and clinical databases, and incorporate clinical or third‐party reports of CSS symptoms, to ensure that research can be generalized across all autistic people. Self‐report can also be limited when discussing bodily sensations and sensory processing differences, as there are individual differences in the ability to perceive internal sensations (interoception) and this can be more pronounced in autistic people (DuBois et al. 2016). Research has found there is often a mismatch between self‐reported interoceptive aptitude and actual performance (Garfinkel et al. 2015), and that this is particularly found in autistic adults (Garfinkel et al. 2016; Quadt, Critchley, and Garfinkel 2018). Future studies exploring the relationship between CSSs and autism could therefore incorporate quantitative sensory testing or other recommended tests for central sensitization such as pressure pain thresholds (den Boer et al. 2021; Neblett et al. 2024).

It should also be noted in the limitations of this study that the questionnaire included a variable for “gender” but not for “sex assigned at birth.” Participants were able to check one of three boxes (male/female/prefer to self‐describe), and then provide further details for the third option. Participants could have interpreted this wording as either “sex assigned at birth” or as “gender of identification,” and therefore there is a chance that some of the participants included in the above analyses may have been transgender. As for our previous study (Grant et al. 2022), participants that ticked the box for “self‐describe,” of whom the majority were non‐binary or agender, had to be excluded due to the small sample size. This was unfortunate, as there is research that indicates gender non‐conforming and trans autistic people may be at increased risk of physical health problems (Argenyi, Mereish, and Watson 2023; Hall et al. 2020; Ryan et al. 2023). Future research should incorporate robust recruitment of trans and non‐binary autistic adults, to identify whether this group experience greater CSS symptoms than cisgender autistic men or women.

Dunn‐Šidák corrections were made to the group differences analyses to minimize the chance of type I errors, since this research is exploratory and in its early stages. It must be noted that use of this statistical method may increase the possibility of type II errors, or false negatives, and this is notable as there were several instances where group differences were observed but were not found to be significant.

It should also be noted that the figure of 145 used as a cut‐off on the Autism Quotient for this study was based on Dutch data and a limited clinical sample (Hoekstra et al. 2008). As very few research studies have used the continuous scoring method of the AQ utilized in this study, this was unavoidable, but use of this cut‐off could potentially over or under‐estimate the relevance of higher autistic traits in CSS only participants. The AQ subscale analysis should also be treated as exploratory and considered alongside research showing that some of the proposed subdomains have low weighting and vary in reliability (Austin 2005; Broadbent, Galic, and Stokes 2013; Hurst et al. 2007).

The questionnaire data used in this study allowed participants to provide details about any clinical health diagnoses, not just CSS conditions, as well as allowing participants to indicate if they suspected that they had a CSS, or autism, but were not diagnosed. We decided to delineate our groups based on clinical diagnosis only, as this allowed us to consider possible diagnostic overshadowing or under‐diagnosis. We recognize these group comparisons did not take into account suspected diagnoses of CSS or autism. Moreover, we focused our study on CSS specifically and did not include clinical diagnoses of other physical health conditions.

Cross‐sectional studies are always limited when exploring cause and effect, and it should be noted that inferences drawn from these analyses, such as through path analyses, must be approached with caution. Longitudinal studies exploring these relationships over a longer period would be beneficial.

## 6 Conclusions

In conclusion, clinicians need to be aware that CSS symptoms and diagnoses are more common in autistic people, and a large crossover in physical health symptoms between autism and CSSs means that there is a risk of diagnostic overshadowing between these two groups. Health professionals should be mindful that physical health symptoms should not be ignored in autistic adults, and that a subset of CSS patients may include people with undiagnosed neurodevelopmental conditions. Furthermore, this study builds on research indicating that the level of sensory sensitivity observed in CSS patients can be equivalent to that in autism, suggesting that environmental adjustments and accommodations offered to autistic people could be equally helpful for those experiencing central sensitisation.

## Author Contributions

S.G. proposed the research question and developed the initial research design. S.G. and R.A.H. prepared and administered the questionnaire, and S.G. managed the corresponding data. S.G. analyzed and interpreted the data with support from R.A.H. and S.N. and wrote the first draft of the manuscript. All authors read, contributed to, and approved the final manuscript.

## Ethics Statement

The protocol of this study was approved by the PNM Research Ethics Subcommittee at King's College London (approval number HR‐18/19–8634) and all participants provided written informed consent.

## Conflicts of Interest

The authors declare no conflicts of interest.

## Data Availability

The datasets collected and analysed in the current study will be made available via the UK Data Service.
